# Macrophage Proinflammatory Responses to Microorganisms and Transplanted Organs

**DOI:** 10.3390/ijms21249669

**Published:** 2020-12-18

**Authors:** Malgorzata Kloc, Ahmed Uosef, Jacek Z. Kubiak, Rafik M. Ghobrial

**Affiliations:** 1 The Houston Methodist Research Institute, Houston, TX 77030, USA; Auosef@houstonmethodist.org (A.U.); RMGhobrial@houstonmethodist.org (R.M.G.); 2 Department of Surgery, The Houston Methodist Hospital, Houston, TX 77030, USA; 3 MD Anderson Cancer Center, Department of Genetics Houston, The University of Texas, Austin, TX 77030, USA; 4 Laboratory of Regenerative Medicine and Cell Biology, Military Institute of Hygiene and Epidemiology (WIHE), 01-163 Warsaw, Poland; jacek.kubiak@univ-rennes1.fr; 5 Cell Cycle Group, Faculty of Medicine, Institute of Genetics and Development of Rennes (IGDR), University Rennes, UMR 6290, CNRS, 35043 Rennes, France

**Keywords:** macrophage, transplantation, infection, chronic rejection

## Abstract

Tissue-resident macrophages and those conscripted from the blood/bone marrow are professional phagocytes. They play a role in tissue homeostasis, replacement, and healing, and are the first-line responders to microbial (viral, bacterial, and fungi) infections. Intrinsic ameboid-type motility allows non-resident macrophages to move to the site of inflammation or injury, where, in response to the inflammatory milieu they perform the anti-microbial and/or tissue repair functions. Depending on the need and the signaling from the surrounding tissue and other immune cells, macrophages acquire morphologically and functionally different phenotypes, which allow them to play either pro-inflammatory or anti-inflammatory functions. As such, the macrophages are also the major players in the rejection of the transplanted organs making an excellent target for the novel anti-rejection therapies in clinical transplantation. In this review, we describe some of the less covered aspects of macrophage response to microbial infection and organ transplantation.

## 1. Types of Macrophages

There are several ways to categorize macrophages. In the broadest sense, macrophages can be categorized into two main groups: 1. the resident macrophages, which derive from the yolk sack and populate given tissue/organ during embryonic development, and 2. the blood/bone marrow-derived macrophages acquired by the tissue/organs after birth [1]. The resident macrophages are usually stationary and self-renewing. The blood/bone marrow-derived macrophages are highly mobile and are recruited into the tissue/organ from the blood depending on the immune response demands. The resident macrophages have been also categorized into different types based on the anatomical location, such as alveolar macrophages (in the lungs) [2], adipose tissue macrophages (in the fat) [3], Kupffer cells (in the liver) [4], red pulp macrophages (in the spleen) [5], peritoneal macrophages (in the peritoneal cavity) [6], Hofbauer cells (in the placenta) [7], tumor-associated macrophages (TAMs) [8], microglia and meningeal macrophages in the CNS [9], and many others [10,11]. The resident and blood-derived macrophages are also categorized based on the function they perform and the molecules and factors they produce, into M0 (monocytes/naïve macrophages), M1 (pro-inflammatory, produce IL-6, IL-12, and TNF- α), M2 (anti-inflammatory, produce Arginase-I, IL-10, and TGF-β), Mreg (regulatory with anti-inflammatory properties, produce interleukin IL-10) [12,13]. Other macrophage types are the recently discovered Mox macrophages that develop in response to oxidative damage and play a role in chronic inflammation [14,15], and M4 macrophages that form in response to the infection with the leprosy bacterium [16]. The M2 macrophages are further divided into M2a, M2b, M2c, and M2d subtypes, based on the signaling they receive and the induced transcriptional response [17,18]. Studies from our laboratory showed that the macrophage phenotype also depends on the mitochondrial functions and ADP/ATP homeostasis [6]. Although all these rigid categories were invented to facilitate our comprehension of macrophage functions, in reality, the macrophages can readily switch properties and functional phenotypes depending on the milieu and signals they receive from the surrounding tissues and other immune cells [19].

Recently, a novel, nerve- and airway-associated macrophage subtype (NAMs) has been identified in humans and mice [20]. NAMs are tissue-resident, self-renewing, and derive from the embryonic yolk sac. They express immunoregulatory genes under normal and inflammatory conditions, and their role is to keep the inflammatory response in check. They rapidly proliferate after influenza virus infection, and their depletion in mice aggravates the virus-induced inflammation of the lungs [20,21]. Because of these properties, NAMs may be very important for the management of the acute respiratory distress syndrome (ARDS) in the lungs of COVID-19 patients, where the exacerbated immune response (cytokine storm) of alveolar macrophages causes fatal damage to the lungs [22]. Below we give a more detailed description of one of these macrophage subtypes of TAMs and summarize how they can be targeted by novel anticancer therapies.

### Tumor-Associated Macrophages (TAMs)

TAMs were discovered in 1970 as predominant immune cells present in the tumors. Although there are many examples of the TAMs’ role in tumor progression, there are also instances of anti-tumor activity of TAMs. For example, in some colorectal tumors, TAMs induce cancer cells to produce more of the inflammatory mediator galectin-3, which recruits additional TAMs to the tumor. Additionally, TAMs release proinflammatory factors IFN-γ, IL-1, and IL-6, which activate T cell response against the tumor. The resulting amplified immune response destroys the cancer cells [8,23]. However, in the majority of cancers, the TAMs have anti-inflammatory and immunosuppressive activities, which by sabotaging the host immune response promote cancer progression, aggressiveness, and metastasis. The recruitment of the monocytes to the tumor and their differentiation into TAMs occurs in response to the cytokines and chemokines, such as the high mobility group box protein 1 (HMGB1) alarmin, CCL2, CCL3, CCL4, CCL5, CCL7, CCL8, CXCL12, VEGF, PDGF, and IL-10, produced by the cells present in the tumor [8,23]. It is well established that the growth of tumors above a few mm requires vascularization through the formation of the new blood vessels (neo-angiogenesis). Studies showed that TAMs initiate and promote angiogenesis through the secretion of VEGF, PDGF, TGF-β), and the FGF growth factors. They also release metalloproteases MMP-1, MMP-2, MMP-3, MMP-9, and MMP-12 and other matrix-degrading and remodeling proteins, which facilitate the sprouting of the new blood vessels. TAMs also promote the formation of lymphatic vessels (lymphangiogenesis), which facilitate tumor spreading [8,23]. Because of these tumor-promoting activities the TAMs became an excellent target for novel anti-cancer therapies [8,24]. One of the biggest problems in cancer treatment is the development of the resistance to therapy. Many studies showed that TAMs interfere with many commonly used anti-cancer therapies, and are involved in the development of the resistance to immune-checkpoint blockade therapy, radiotherapy, chemotherapy, and anti-angiogenic therapy. These TAMs’ properties require designing novel therapies, which either reprogram TAMs from the pro-tumorigenic to anti-tumorigenic functions, or directly kill TAMs, or inhibit their recruitment into the tumor [24]. Various chemotherapeutics, such as trabectedin (Yondelis) and bisphosphonates with preferential anti-macrophage toxicity are used to eliminate or reduce the number of TAMs. The therapies using various antibodies or kinase inhibitors inhibit the CCL2/CCR2-, CXCL12/CXCR4-t, or CSF-1/CSF-1R-dependent monocyte/macrophage recruitment into the tumor. TAMs reprogramming therapies use the agonists, such as poly I:C, imiquimod (R837), or Resiquimod (R848), of Toll-like receptors (TLRs), which activate macrophage polarization pathway, to revert TAMs toward the anti-tumor activity. In recent years the delivery of mRNA, siRNA, or miRNA has been used as the TAMs reprogramming therapies [24]. These RNAs either silence the expression of the chosen genes regulating the immunosuppressive activities or upregulate the expression of pro-inflammatory factors in TAMs [24]. Anfray et al. [24] give a comprehensive list of the currently used anti-TAMs therapies in different types of cancer.

## 2. Macrophage Response to Microorganisms

Besides the production of the factors that signal other immune cells to fight the infection, macrophages employ two main strategies to fight the invading microorganisms: 1. phagocytosis followed by the destruction of the pathogen or 2. depletion of factors essential for pathogen survival and replication [25,26]. Recent studies show studies the existence of the third microbicidal mechanism- the macrophage extracellular traps [27,28].

**Figure 1:**
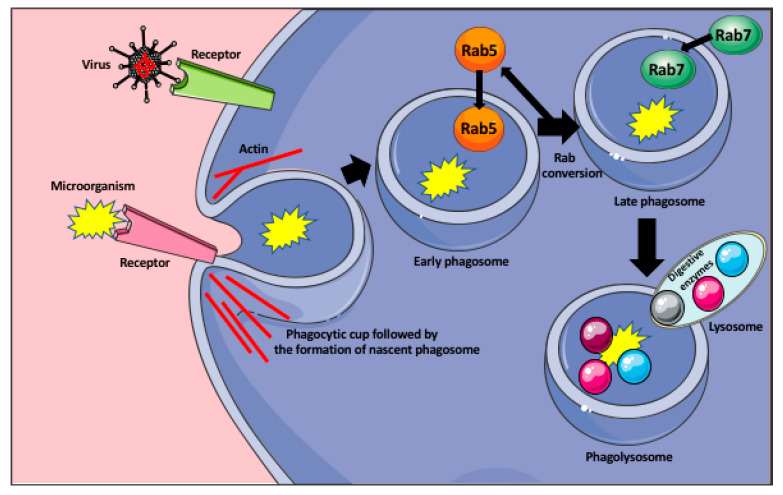
Phagocytosis of microorganisms by macrophage. Recognition of the microorganism (bacteria, or virus) by the appropriate receptor induces the formation of the phagocytic cup that engulfs the microorganism and pinches off the membrane as a nascent phagosome. The formation of the phagocytic cup and detachment of the phagosome from the membrane are actin dependent. The nascent phagosome recruits small GTPase Rab5 that induce its remodeling and becomes the early phagosome. In the process of the Rab conversion, the early phagosome loses Rab5 and acquires Rab7 becoming the late phagosome. The Rab7 induces signaling pathways allowing the late phagosome to fuse with the lysosomes, containing various digestive enzymes, and become the phagolysosome that degrades the microorganism.

To locate the invading microorganisms, the macrophages send out long, actin-rich protrusions (pseudopodia), which probe the extracellular space for the presence of pathogens and phagocytic targets [26,29]. Phagocytosis is initiated by the macrophage membrane receptors, which recognize bacterial membrane protein or the components of the virus, or/and serum opsonins, such as IgG or complement proteins, which are produced by the immune cells upon recognition of a foreign antigen. Following the recognition, they coat the pathogens and mark them for phagocytosis and further immune response [26]. The recognition of the microorganisms occurs through the variety of the cellular pattern recognition receptors (PRRs), such as Toll-like receptors (TLRs), C-type lectin-like receptors (CLRs), nucleotide-binding and oligomerization domain-like receptors (NLRs), cytoplasmic double-stranded DNA (dsDNA) receptors, and RIG-I-like receptors (RLR). They recognize the pattern- or danger-associated molecular patterns (PAMPs od DAMPs) [30]. The target recognition by macrophage receptors initiates signaling pathways, which trigger macrophage membrane remodeling and formation of the vesicle (a nascent phagosome) that encloses the target and moves it inside the macrophage cytoplasm. The nascent phagosome interior does not have any mechanisms to kill the pathogen and has to go through successive steps of maturation to become microbicidal. First, the nascent phagosome becomes the early phagosome through the recruitment of the small GTPase Rab5, which induces phagosome remodeling. Next, in the process called the Rab conversion, the early phagosome loses Rab5 and acquires Rab7 protein becoming the late phagosome [26,31]. The Rab7 induces signaling pathways allowing the late phagosome to fuse with the lysosomes to become the phagolysosome. All these steps are accompanied by the progressive acidification of the phagosome interior, through its vacuolar ATPase (v-ATPase) that pumps H^+^ from the macrophage cytoplasm to the phagosome [26,32]. The interior of the phagolysosome is highly acidic (≤ pH 5) and contains lipases, proteases, nuclease, glycosidases, and phosphatases, which degrade and kill the ingested microbe (Figure 1) [26]. Additionally, the phagosomes also activate the NADPH oxidase (Nox2), and oxide synthase 2 (Nos2), which catalyze the formation of reactive oxygen and nitrogen species highly toxic for the microbes [26]. One of the examples of how macrophages suppress viral replication and prevent immunopathological changes in the infected organ, is the activity of the liver macrophages, the Kupffer cells during viral infection. In humans, a persistent infection with hepatitis B or C viruses causes liver damage, cirrhosis, hepatocellular cancer, and eventually, liver failure. The majority of the liver damage is not caused directly by the viruses but by the aggressive response (secretion of INF γ and perforin) of CD8^+^ T cells against the infected hepatocytes [33,34,35]. Lang et al. [35] studied the role of liver Kupffer cells in the inhibition of virus dissemination and prevention of liver damage. They showed that in lymphocytic choriomeningitis virus (LCMV strain WE) infected mice, the depletion of liver macrophages with clodronate-filled liposomes, resulted in severe liver damage. They showed that the liver Kupffer cells phagocytose the virus within 10–60 min after the intravenous LCMV infection. They not only capture the virus but also prevent its replication and dissemination in the IFN-I-dependent manner [35]. However, in contrast to mice, the exact role of human Kupffer cells in the control of viral infection of the liver is still not fully understood and requires further studies.

Macrophages can also sequester micro-elements necessary for microbe survival. This process is called “nutritional immunity” [26]. The phagosomes contain the natural resistance-associated macrophage protein 1 (NRAMP-1) that removes the Fe and Mn from the phagosome lumen and away from the engulfed microorganism, to the cytosol, where they bind to the chaperon proteins that deliver them to the storage proteins, such as ferritin and calprotectin [26,36].

**Figure 2:**
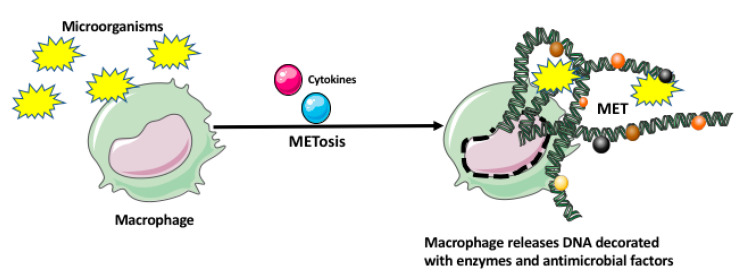
Macrophage extracellular trap (MET) formation by macrophage. The microorganisms and/or various cytokines may induce a unique cell death program in the macrophage, called METosis. The METosis causes breakage of the macrophage nuclear envelope and release of strands of DNA, which form the MET decorated with the enzymes and antimicrobial factors.

Recently, it has been also shown that macrophages, similar to neutrophils, eosinophils, basophils, and mast cells can immobilize and kill microorganisms by entangling them within the extracellular traps (ETs) [27,28]. The macrophage extracellular traps (METs) are produced in response to the microorganisms and/or cytokines, which induce a unique cell death program, called METosis. The METosis causes breakage of macrophage nuclear envelope and release of strands of DNA, which form a fibrillar network decorated with hypercitrullinated histones, antimicrobial proteins and peptides, and enzymes (metalloproteinases, myeloperoxidase, lysozyme, lactoferrin, elastase) (Figure 2) [27,28]. Aggregation of various antimicrobial proteins on the DNA strands of METS is facilitated by their high affinity for DNA. Additionally, the other macrophages and immune cells can sense DNA and proteins present in the METs, which leads to the production of various proinflammatory mediators.

Below we describe how bacteria and viruses can elude macrophages and even force them to become a habitable host or a disseminating tool.

### 2.1. How Bacteria Evade Macrophages

The macrophages and other immune cells recognize the pathogen-associated molecular patterns (PAMPs) of the pathogen through the various pattern recognition receptors (PRRs) [30,37]. One of the evasion methods used by the microorganisms is the change of the antigenic properties of the surface to more antigenically neutral and less recognizable by the immune system. The cell surface of prokaryotes is covered by the polysaccharide capsule that is attached to the cell surface by lipid A or phospholipid, which are recognized by the PRRs. Some bacteria synthesize the modified versions of Lipid A, which are non- or poorly recognizable by the pattern recognition receptors [30,38,39,40]. If still recognized, the bacteria can try to avoid phagocytosis. Because macrophages prefer to phagocyte smaller targets, one of the ways bacteria, and other pathogenic microorganisms, can evade phagocytosis is to increase the size. It has been shown that *C. neoformans* yeast and many species of *Mycobacteria* increase the size to escape phagocytosis [41]. Size increase can be either achieved by the aggregation of bacteria into multi-bacterial biofilms, delaying the division, and/or changing the rate of metabolism to increase the individual cell mass. If the bacteria have not been able to escape phagocytosis, they still can try to survive inside the acidic environment of the phagosome by developing the tolerance to acidity. It has been shown that *Mycobacteria* can survive and grow inside the phagolysosome and that the molecule, which confers acid tolerance is the mycobacterial serine protease Rv3671c (MarP), [42]. Some bacteria can develop resistance to the antibacterial drugs only when they are inside the macrophage; the depletion of host macrophages decreases bacterial drug tolerance. The macrophage environment changes the bacteria cell cycle and functions allowing them to pump out the drug [42]. Once the microorganisms such as bacteria and fungi successfully evaded recognition and destruction, and/or found the immune non-responsive macrophages, they can use macrophage as a protective niche against the other immune cells with higher microbicidal activity, or temporary housing where they grow, replicate or germinate before the non-lytic exit from the host cells, and dissemination to other cells, tissues and, organs. The examples include *Burkholderia cenocepacia* (an opportunistic pathogen causing infections in immune-compromised humans), *Candida albicans* (nonharmful yeast that can cause infection after entering the bloodstream or the internal organs), and *Cryptococcus neoformans* (an environmentally ubiquitous fungus that can infect lungs and nervous system in people with the weakened immune system) [41].

### 2.2. Macrophage Role in Virus Dissemination

Despite being a major virus elimination tool of the immune system, under certain conditions, macrophages can be infected by the virus, and, by becoming the virus repository, spread the virus, and exacerbate the infection. This requires the virus to assure the longevity of the host macrophages (by turning off the cell death program) and reprogramming or escaping their anti-viral response [43]. Klepper and Branch [44] list the mutations in many human and animal viruses, such as influenza virus, rabies virus, dengue, lymphocytic choriomeningitis virus (LCMV), feline coronaviruses (FCoV), Theiler’s murine encephalomyelitis virus (TMEV), human immunodeficiency virus type-1 (HIV-1), and human cytomegalovirus (HCMV), which give them ability to infect and replicate in the macrophages. Currently, over 35 types of viruses, belonging to 13 different families, have been shown to infect and disseminate through monocytes and/or macrophages [45]. Recent studies of the possible induction of diabetes mellitus by the SARS-CoV-2 suggest that SARS-CoV-2-infected monocytes/macrophages may deliver and spread the virus to the pancreas, damaging the pancreatic islets and β-cells [46].

## 3. The Specificity of Macrophage Response

Although the macrophage response to any infection fits the described above general formula, it greatly varies depending on the type/species of the invading microorganism and the tissue/organ it targets. Below we describe a few examples of such specific responses.

### 3.1. Fungal Infection

Infection with the opportunistic fungi such as *Pneumocystis*, *Aspergillus*, and *Cryptococcus* may cause life-threatening disease in the lungs (pneumonia, pulmonary aspergillosis, bronchopulmonary aspergillosis (ABPA) in patients with severe asthma or cystic fibrosis, and cryptococcosis, respectively) [47,48]. Although the main target of these fungi is the lungs, some of them can also infect the brain and/or the central nervous system, causing, for example, Cryptococcal meningoencephalitis and neuroaspergillosis. Such fungal infections are especially common in the immune-suppressed HIV/AIDS patients, cancer patients receiving chemotherapy or patients after transplantation who receive anti-rejection immunosuppressive therapies [47,49].

Characteristic for a specific fungus molecules belonging to the pathogen-associated molecular patterns (PAMPs) are recognized by the macrophages (or/and other immune cells of the innate immune system) by the pattern recognition receptors (PRRs), such as C-type lectin receptors (CLRs), Toll-like receptors (TLRs), and NOD-like receptors (NLRs). For example, *Aspergillus fumigatus* is recognized by Dectin-1 and TLR receptors [50]. These authors showed that the alveolar macrophages from Dectin-1- deficient mice showed impaired fungal uptake. Binding of the fungus to one (or several) of these receptors present on the macrophage surface activates a cascade of PAMP-response signaling, such as, for example, the authophagy pathway. Bhatia et al. [50] showed that the infection with *A. fumigatus* induces alveolar macrophages to express Arginase 1 (Arg1), that is a marker of M2 macrophages, a novel mammalian lectin Ym1, and mannose receptor C type 1 (MRC1) CD206. They concluded that, at least in mice, the M2 macrophages are crucial players in defense against *A. fumigatus* infection [50].

Studies of the Pneumocystis pneumonia (PCP) caused by the opportunistic fungus *Pneumocystis jirovecii* showed that the main antigen of Pneumocystis is a heavily mannose-glycosylated major surface glycoprotein (MSG, also called the gpA). Thus, this antigen is readily recognized by the mannose receptor (MR) present at the surface of alveolar macrophages [51]. Binding of *Pneumocystis* to MR leads to the activation of the NF-κB pathway resulting in the expression of matrix metalloproteinase-9, and the pro-inflammatory IL-8 [52]. Other studies showed that another antigen present in the *Pneumocystis* cell wall is the β-glucan, Dectin-1, which is recognized by macrophage PRR receptors. In mice, binding of β-glucan induces alveolar macrophages to synthesize TNF-α macrophage inflammatory protein (MIP)-2 (the murine equivalent of IL-8) through the activation of NF-κB signaling [53]. It was further shown that dectin-1 mediates the production of reactive oxygen species [54] that kill the fungus. The ability to produce ROS and, thus, fight the infection was eliminated in dectin-1-knockout macrophages [55].

### 3.2. Mycobacterium Tuberculosis Complex Infection

The *Mycobacterium tuberculosis* complex consists of the genetically similar mycobacteria species (*M. tuberculosis*, *Mycobacterium canettii*, *Mycobacterium africanum*, *Mycobacterium microti*, *M. bovis*, *Mycobacterium caprae*, and *Mycobacterium pinnipedii*) that cause tuberculosis in humans and animals. The major components of Mycobacteria cell wall are specific lipoproteins (Lpps), lipoglycans and complex lipids, which specifically modulate host macrophage response [56]. Mycobacteria evolved several mechanisms, which counteract the microbicidal response of macrophages. One of the avoidance mechanisms is the ability to arrest phagosome maturation by preventing phagosome acidification and fusion with lysosomes to form a mature phagolysosome. It has been shown that Mycobacteria produce the Ndk, a nucleoside diphosphate kinase with ATP/GTP binding activity and hydrolytic activity, which after release from the bacteria accesses the cytosolic surface of the phagosome and prevents its fusion with the lysosomes. Among many different molecules playing a role in the arrest of the phagosome maturation is the tyrosine phosphatase PtpA, which binds to macrophage vacuolar ATP H^+^ pump preventing phagosome acidification [56]. Another counteracting mechanism is the inhibition of macrophage cell death program resulting in the survival of the infected macrophages and dissemination of the Mycobacteria. Several mycobacterial anti-apoptotic genes (nuoG, katG, sodA/secA2, pknE, and Rv3654c/Rv3655c) have been identified in M. tuberculosis. These genes control the production of ROS, the known triggers of macrophage apoptosis [57]. The third avoidance mechanism is the resistance of mycobacteria to the toxic molecules produced by the host. One of the mechanisms involved here is the production of superoxide dismutase enzyme, which counteracts and detoxifies the reactive oxygen (ROS) species produced by the macrophages. Another mechanism involves the synthesis of KatG catalase-peroxidase enzyme KaG that degrades H_2_O_2_ and organic peroxides, and a thiol peroxidase enzyme TpX that catalyze the reduction of hydroperoxides and peroxynitrite, thus counteracting the reactive oxygen (ROS) and nitrogen (RNS) species produced by the macrophages in response to the infection [56].

### 3.3. Mycobacterium Leprae Infection

*Mycobacterium leprae* infection causes leprosy that, depending on the immune response of the patient presents different clinical forms. It can affect skin, peripheral nerves, eyes, respiratory tract, muscle, bone, and testes [58]. The recognition of *Mycobacteria* occurs mainly through the TLRs (TLR1/2) receptors. A genome-wide analysis of *M. leprae* identified 31 lipoproteins which can be potentially recognized by TLR2/1 receptors [58]. There is a correlation between the spectrum of clinical forms of leprosy and the intensity of immune response [59]. Tuberculoid leprosy (T-lep) is characterized by a strong immune response, a high number of M1 macrophages, and a low number of *Mycobacteria*.

In contrast, lepromatous leprosy (L-lep) is characterized by a nigh number of mycobacteria-laden foamy M2 macrophages. The T-lep patients produce a high level of pro-inflammatory factors, such as IFN-γ, TNF, and IL-15, while L-lep patients produce anti-inflammatory cytokines IL-4, IL-10, and IL-13 [60]. The foamy macrophages present in the L-lep lesions are positive for the adipose differentiation-related protein (ADPR). Because the ADPR is a marker of the macrophage lipid load and facilitates fatty acid uptake, it may explain the “foamy” appearance of the macrophages and suggests that *M. leprae* induces lipid accumulation in the macrophages. Such lipid-laden macrophages may have diminished phagocytotic activity and/or undergo apoptosis. The cytokine anti- or pro-inflammatory profile positively or negatively regulates macrophage autophagocytosis. In the process of autophagocytosis, the macrophage encloses defective organelles or intracellular (invading) microorganisms within the double-membrane vesicles (autophagosomes), which subsequently fuse with the lysosomes to degrade autophagosome content [60]. Recent studies indicate that in the infected macrophages, the *Mycobacteria* are targeted to autophagosomes and that autophagy is differentially regulated in T-lep and L-lep patients [60]. Further studies showed that the skin of lepromatous patients has both M1 and M2 macrophages, with the continuum of different subtypes between, and the dendritic cells [58]. The macrophages are the major responders to *M. leprae* infection, but their phenotype/activity differs depending on the clinical form and targeted organ. For example, the macrophages in the lepromatous skin but not in the tuberculoid (a milder form of leprosy) lesions express a high level of Galectin-3, which plays a role in macrophage and T cell activation [58]. Studies also showed that the macrophages present in lepromatous skin upregulate IL-27 that may activate IFN-β and IL-10, which block the antimicrobial response [58,61]. Although lepromatous macrophages express not only many of the M2 markers (bacterial immune-osensor CD163, indoleamine 2,3 dioxygenase (IDO) that suppresses T and NK cells, generate Tregs, and myeloid-derived suppressor cells), arginase, and steroid receptor RNA activator 1 (SRA-I), but also have some features of M1 macrophages, such as low expression of the iron exporter ferroportin (Fpn-1), which leads to the elevated iron content and may increase *M. leprae* survival [58].

### 3.4. Brain-Eating Amoeba Naegleria Fowleri Infection

*Naegleria fowleri* is a free-living amoeba (FLA) abundant in freshwater and soil that can very rarely (147 patients have been diagnosed in the United States between 1962 and 2020, with only 3 survivals) infect humans and cause lethal primary amoebic meningoencephalitis (PAM) [62,63]. *N. fowleri* has three different stages (forms): a dormant cyst, a migratory flagellate, and the trophozoite that can divide, feed and infect humans. The *Naegleria* trophozoite may enter the human body through the nasal cavity during water-related activities. After attaching to the nasal mucosa, and crossing the olfactory epithelium, it travels along the olfactory nerves (the cranial nerves that conduit sensory smell information) to the olfactory bulbs (a part of the forebrain responsible for smell sensing located just above the nasal cavity) of the brain cerebral hemispheres. Traveling along the nerves allows bypassing the central nervous system barrier. *Naegleria* causes brain damage through direct and indirect effects. Amoeba directly damages the tissue by ingesting the fragments of the tissue (trogocytosis) using the food cup. The food cup forms when the edges of the amoeba pseudopodia come close together surrounding the food material. Another direct effect is the release of cytolytic factors such as neuraminidases, phospholipases, phospholipolytic enzymes, and hydrolases, which cause brain tissue damage. Additionally, upon arriving at the olfactory bulbs, amoeba induces a very intense immune response by the immune cells (including neutrophils and macrophages) that injures the brain tissue [62]. Interestingly, from the still unknown reasons, the detection of the amoebas by the immune system occurs quite late (3–4 days) after the infection, when the amoebas are already in the brain. During the first several hours after the infection, when the amoebas migrate in the host, the innate immune response is very week. Eventually, after amoebas are detected, they illicit a massive influx of the immune cells (neutrophils, eosinophils, monocytes/macrophages), which cause lytic necrosis and hemorrhage of the brain. This delayed detection and lack of early immune response may be responsible for the fatality of the disease [62]. In contrast to the viral and bacterial pathogens (prokaryotes), the eukaryotic amoebas are usually not recognized by the majority of human pattern recognition receptors (PRRs). Instead, the immune system relies on complement activation followed by complement-mediated lysis [63]. Kim et al. [64] studied the effect of *Naegleria* on the macrophage activity in the in vitro model. They found that after 3 h of noncontact co-culturing of the human macrophages (THP-1 cell line) with *Naegleria* trophozoites activated the formation of the NOD-, LRR- and pyrin domain-containing protein 3/Apoptosis-associated speck-like protein containing a CARD (NLRP3/ASC) inflammasomes. The NLRP3 is an intracellular sensor that detects various microbial components, and endogenous and exogenous danger signals, and induces the formation and activation of the NLRP3 inflammasome. The NLRP3 inflammasome is the multimeric complex that activates caspase-1, which in turn controls cleavage-dependent maturation of Interleukin-1β and IL-18 [65,66]. Thus, the *Naegleria* infection induces the inflammasome, caspase-1, inflammatory cytokines, and inflammatory response of the macrophages.

## 4. Macrophage Response to Organ Transplantation

Transplantation of any organ not deriving from the genetically identical tween mounts in the transplant recipient the vigorous immune response against the genetically different transplant. The success of transplantation, and the fitness and survival of the transplant, depend on the therapeutic suppression of the host immune response and, participating in the rejection, immune cells. Transplanted organs undergo the hyperacute rejection that occurs a few minutes after transplantation when the donor and recipient are completely genetically unmatched. The progress in genetic matching nearly eliminated this type of rejection in clinical transplantation. In contemporary transplantation, the transplanted organs undergo two main types of rejection: acute rejection which occurs between the first week and 3 months post-transplantation, and chronic rejection which develops and progresses within many months or years post-transplantation [67,68]. Acute rejection is mainly driven by T cells, with some participation of macrophages, while chronic rejection mainly depends on the macrophages. Macrophages participating in the acute rejection belong to the M1 and M2-like subtypes. They secrete inflammatory factors such as IL-1β, IL-12, IL-18, TNF-α, and IFN-γ, which either directly damage the graft tissues or/and activate endothelial cells and induce the cytotoxic T-cells. They also produce reactive oxygen (ROS) and reactive nitrogen species (RNS), which damage graft and enhance acute rejection [68].

While in present-day transplantation the acute rejection is manageable through the application of immunosuppressive drugs targeting T cell activation, such as cyclosporin (CA), the chronic rejection remains unmanageable and is responsible for a long-term organ failure in clinical transplantation [69]. Chronic rejection causes occlusion of the transplant blood vessels and tissue fibrosis. Both of these processes are regulated by the resident and infiltrating macrophages in response to the inflammatory signals released by the graft. The recruited and resident macrophages stimulate the over-proliferation of the muscle cells in the blood vessel wall which results in the constriction of the vessel lumens, and eventually their complete blockage and the starvation of the graft [67,69,70]. They also stimulate fibrocytes to overproduce the fibrotic factors, leading to a destruction of the graft architecture and integrity [67,69]. Studies from our laboratory showed that macrophage phenotype and movement to the graft are regulated by the small GTPase RhoA pathway and its effector, the actin cytoskeleton that besides the movement, regulates phagocytosis and receptor recycling [71,72,73,74,75]. These findings suggested that targeting the macrophages and RhoA pathway in the graft recipient could decelerate or eliminate chronic rejection of transplanted organs.

**Figure 3:**
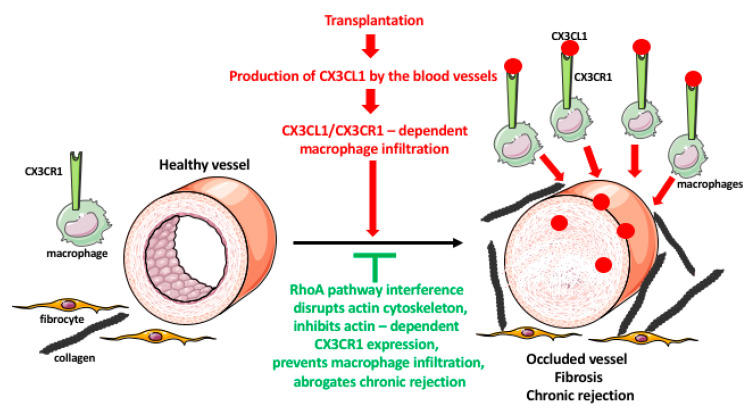
Role of macrophages in transplant rejection. Organ transplantation induces immune responses in the recipient. One of the responses is a massive production of the chemokine fractalkine (CX3CL1) by the endothelial cells of the graft blood vessels. The CX3CL1 recruits the monocytes/macrophages, which express the fractalkine receptor (CX3CR1), from the blood to the vicinity of the blood vessels. The macrophages induce an over-proliferation of the smooth muscle cells in the blood vessel wall, and fibroblast/fibrocyte to express a huge quantity of fibrotic factors such as collagen. These result in the occlusion of the blood vessel lumen, and graft tissue fibrosis, leading to chronic rejection of the transplant. The expression and recycling of the macrophage receptors are actin-dependent, and actin is regulated by the RhoA pathway. The interference (either RhoA deletion or pharmacologic inhibition) with the RhoA pathway disrupts the normal functioning of actin filaments and actin-dependent processes such as receptor expression and recycling. The lowered expression of CX3CR1 receptors makes macrophages less or nonresponsive to the fractalkine, prevents their infiltration into the graft, and inhibits chronic rejection.

Indeed, our studies in the rodent cardiac transplantation models showed that the genetic interference with the RhoA pathway in the transplant recipients inhibits macrophage infiltration of the graft and abrogates chronic rejection [76]. The inflammatory processes in the graft induce endothelial cells of the graft blood vessel to secrete fractalkine (CX3CL1) chemokine that recruits macrophages, which express the fractalkine receptor (CX3CR1), to the vicinity of the blood vessels. Once there, the macrophages induce the over-proliferation of the smooth muscle cells of the vessel wall and fibrosis of the surrounding tissues. These lead to the occlusion of the blood vessel lumen, destruction of tissue integrity, and chronic rejection of the graft. We showed that macrophage-specific knockout of RhoA decreases the level of CX3CR1 receptors on the macrophage surface resulting in the under-responsiveness of macrophage to fractalkine signaling and reducing macrophage entry into the graft. This, in turn, leads to the lessening of vessel occlusion and fibrosis, and inhibition of chronic rejection of the graft [76]. Proper recycling and expression of the receptors at the cell surface depends on the endocytic/exocytic vesicular pathway, which is actin/RhoA dependent. Accordingly, we also showed that the decrease in the level of CX3CR1 receptors in the RhoA-deleted macrophages resulted from the disruption of the actin cytoskeleton and defective vesicular recycling of the receptors [76]. We also showed that the disruption of the RhoA pathway by the inhibitors of the downstream effector of RhoA, ROCK kinase, or by inhibitors of the upstream regulators of RhoA, GEFs disrupts macrophage actin and affects their shape, motility, and functional phenotype [75]. Our tests of many commercially available inhibitors (Y27632, Azaindole-1, Fasudil, SAR-407899, and SLX-2119) of the RhoA/ROCK pathway showed that except SAR-407899 and SLX-2119 (which only inhibit fibrosis) they are highly effective in inhibiting macrophage influx and chronic rejection of rat and mouse cardiac allografts (Figure 3) [71,77]. In our search for the clinically approved RhoA inhibitors, we found that Fingolimod (FTY720) and Siponimod, which are used for the treatment of multiple sclerosis [78,79,80,81], also inhibit RhoA, expression of CX3CR1 receptors, macrophage infiltration, and chronic rejection of rodent cardiac allografts [81]. These findings will allow repurposing these multiple sclerosis drugs to clinical transplantation.

## 5. Conclusions

In this review, we described phagocytic response of macrophages to microbial infection and macrophage involvement in the chronic rejection of transplanted organs. We have chosen these particular topics in seemingly unrelated arms of the immune response as the example of two, out of the myriad, of very diverse macrophage activities and functions.

The macrophages seem to be an excellent target for the development of novel therapies against infectious diseases and transplant rejection. For example, pharmaceutical targeting of macrophage movement could prevent infiltration of the transplanted organs and subsequent chronic rejection, and in the viral infections, prevent the dissemination of viruses to various organs.
